# Conventional and Advanced Imaging Techniques in Post-treatment Glioma Imaging

**DOI:** 10.3389/fradi.2022.883293

**Published:** 2022-06-28

**Authors:** Anna Y. Li, Michael Iv

**Affiliations:** ^1^Department of Radiology, Stanford University School of Medicine, Stanford, CA, United States; ^2^Division of Neuroimaging and Neurointervention, Department of Radiology, Stanford University School of Medicine, Stanford, CA, United States

**Keywords:** advanced imaging, perfusion MRI, fractional tumor burden, post-treatment, glioma

## Abstract

Despite decades of advancement in the diagnosis and therapy of gliomas, the most malignant primary brain tumors, the overall survival rate is still dismal, and their post-treatment imaging appearance remains very challenging to interpret. Since the limitations of conventional magnetic resonance imaging (MRI) in the distinction between recurrence and treatment effect have been recognized, a variety of advanced MR and functional imaging techniques including diffusion-weighted imaging (DWI), diffusion tensor imaging (DTI), perfusion-weighted imaging (PWI), MR spectroscopy (MRS), as well as a variety of radiotracers for single photon emission computed tomography (SPECT) and positron emission tomography (PET) have been investigated for this indication along with voxel-based and more quantitative analytical methods in recent years. Machine learning and radiomics approaches in recent years have shown promise in distinguishing between recurrence and treatment effect as well as improving prognostication in a malignancy with a very short life expectancy. This review provides a comprehensive overview of the conventional and advanced imaging techniques with the potential to differentiate recurrence from treatment effect and includes updates in the state-of-the-art in advanced imaging with a brief overview of emerging experimental techniques. A series of representative cases are provided to illustrate the synthesis of conventional and advanced imaging with the clinical context which informs the radiologic evaluation of gliomas in the post-treatment setting.

## Introduction

Gliomas are the most commonly occurring malignant primary central nervous system (CNS) tumor in adults. Glioblastoma (GBM) is the most common malignant brain tumor in adults with an incidence of 14.3% of all CNS tumors and 49.1% of malignant CNS tumors (1). Despite advancements in diagnostic and therapeutic techniques over the last few decades, the prognosis of these patients remains dismal with a median overall survival (OS) of <5 years for anaplastic glioma and ~15 months for GBM (1, 2). The current standard of care for GBM consists of maximal safe resection followed by chemoradiotherapy (CRT) including concurrent and adjuvant temozolomide (TMZ) chemotherapy (2–4). Anti-angiogenic therapy has become a critical treatment approach in the treatment of recurrent gliomas (4, 5). In particular, bevacizumab (BVZ), a monoclonal antibody to vascular endothelial growth factor (VEGF), which was approved by the Food and Drug Administration (FDA) in 2009, has resulted in pharmacological debulking and volumetric tumor reduction leading to neurologic palliation and increased progression free survival (PFS) although there has been no significant improvement in OS (5–7).

Glioma tumor biology is marked by neovascularization with resultant tumor progression and therapy resistance (2, 8, 9). Specifically, tumor cells are prone to infiltration of the normal brain parenchyma with remnant tumor stem cells after resection and CRT, which is a source of tumor recurrence (2, 8–10). Tumor heterogeneity is a hallmark of gliomas in the post-treatment setting as there is often an admixture of treated and viable tumor cells and treatment-induced necrosis (8, 11). Glioma-associated vessels demonstrate marked spatial heterogeneity, and the marginal tumor area consists of proliferative and invasive cells with increased microvascular density and active angiogenesis (2, 9). Reduced vascular perfusion with compressed and tortuous vascular networks is seen in the lesion core which results in tissue hypoxia and necrosis (2, 9). Each of these essential aspects of glioma pathophysiology and tumor biology has salient ramifications in anatomic, metabolic, and functional imaging of gliomas in the post-treatment setting.

**Figure 1:**
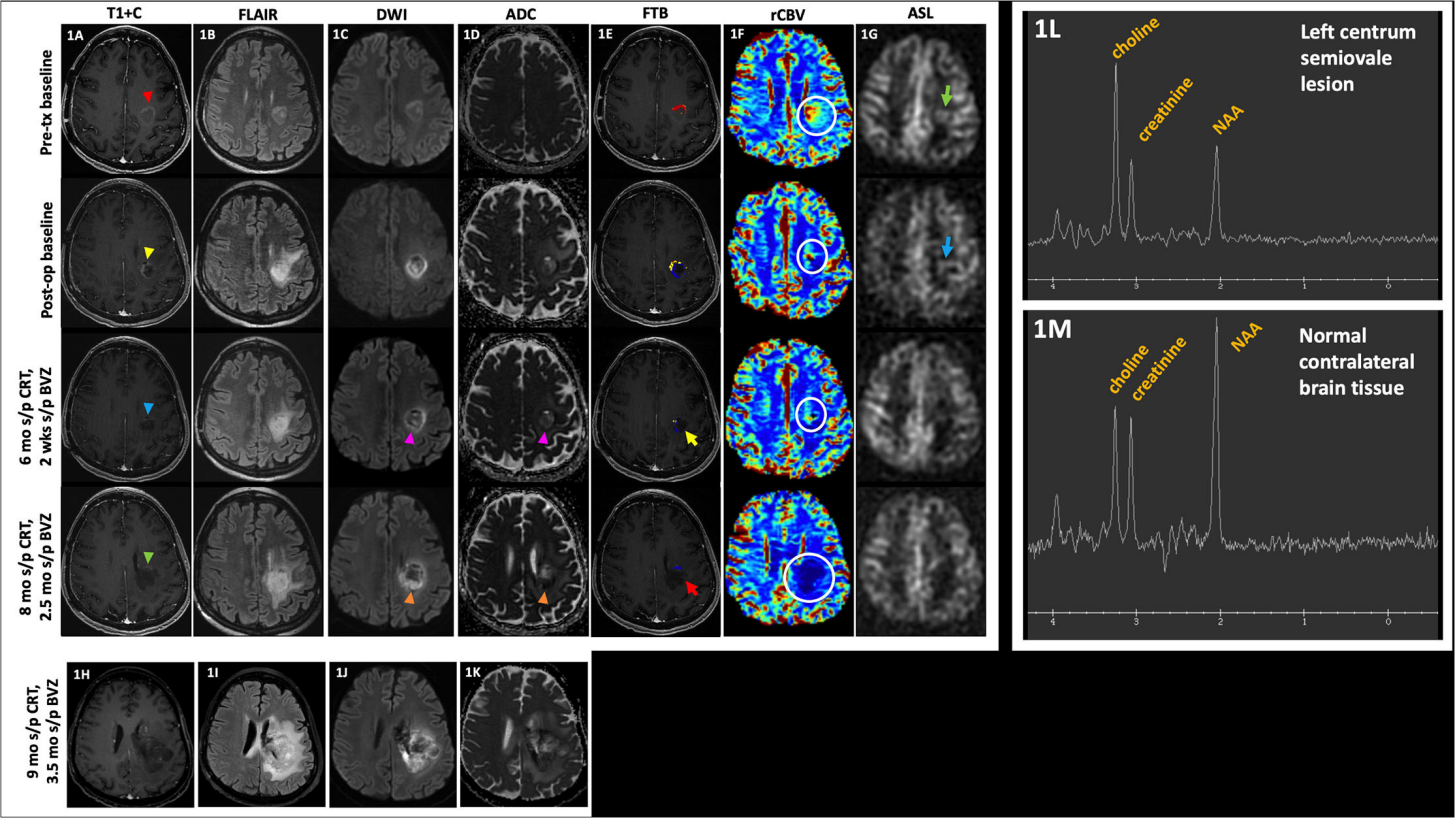
Pseudoresponse. A 57 year-old female with anaplastic astrocytoma (IDH wt, MGMT-) initially demonstrated a mildly enhancing left posterior frontal lobe mass [**(A)**, top row, red arrowhead], with FLAIR hyperintensity [**(B)**, top row], mild diffusion restriction [**(C,D)**, top row], small area of high fractional tumor burden (FTB_high_) at the anterior aspect of the lesion [**(E)**, top row], increased rCBV [**(F)**, top row, white circle], and ASL hyperperfusion [**(G)**, top row, green arrow]. After resection, there is mild residual enhancement at the anteromedial aspect of the lesion [**(A)**, 2nd row, yellow arrowhead] with increased perilesional FLAIR hyperintensity [**(B)**, 2nd row], increased diffusion restriction [**(C,D)**, 2nd row], decreased area of FTB_high_ at the anterior aspect of the lesion [**(E)**, 2nd row], decreased rCBV [**(F)**, 2nd row, white circle], and mild ASL hyperperfusion [**(G)**, 2nd row, blue arrow]. The patient was then initiated on BVZ. At 6 mo s/p CRT and 2 wks s/p BVZ, there is decreased enhancement [**(A)**, 3rd row, blue arrowhead], mildly decreased but persistent FLAIR hyperintensity [**(B)**, 3rd row], decreased diffusion restriction [**(C,D)**, 3rd row, pink arrowheads], decreased area of high fractional tumor burden (FTB_high_) [**(E)**, 3rd row, yellow arrow], mildly decreased rCBV [**(F)**, 3rd row, white circle], and no substantial ASL hyperperfusion [**(G)**, 3rd row]. At 8 mo s/p CRT and 2.5 mo s/p BVZ, there is enlargement of the hypoenhancing lesion [**(A)**, 4th row, green arrowhead] with increased perilesional FLAIR hyperintensity [**(B)**, 4th row], and increased diffusion restriction [**(C,D)**, 4th row, orange arrowheads]; however, there is persistently low FTB_high_ [**(E)**, 4th row, red arrow] and rCBV [**(F)**, 4th row, white circle], likely due to normalization of tumor blood vessels and perfusion in the setting of BVZ. After BVZ was initiated, enhancement decreased with diffusion restriction although the FLAIR hyperintensity persisted. Subsequent outside hospital MR 9 mo s/p CRT showed increased hypoenhancing tumor **(H)** with markedly increased FLAIR hyperintensity **(I)** and diffusion restriction **(J,K)**. These findings are compatible with pseudoresponse (i.e., progressive infiltrative pattern of non-enhancing tumor) that is seen after BVZ therapy. Of note, MRS performed on her baseline pre-tx study showed the classic MRS glioma profile of increased choline, decreased creatinine, and decreased NAA peaks **(L)** when compared to the contralateral normal brain tissue **(M)**. MRS was not performed on subsequent follow-up studies. ADC, apparent diffusion coefficient; ASL, arterial spin labeling; BVZ, bevacizumab; CRT, chemoradiotherapy; FLAIR, fluid-attenuated inversion recovery; FTB, fractional tumor burden; IDH, isocitrate dehydrogenase; K^trans^, volume transfer coefficient; MGMT, O-6-methylguanine-DNA methyltransferase; mo, months; MRS, MR spectroscopy; op, operative; rCBV, relative cerebral blood volume; s/p, status post; T1+C, post-contrast T1-weighted images; tx, treatment; wks, weeks; wt, wildtype.

Several pertinent clinical and radiological phenomena in the post-treatment imaging of gliomas deserve discussion. Approximately 30% of glioma patients who receive CRT may develop new increasing areas of contrast-enhancement and peritumoral T2/fluid attenuated inversion recovery (FLAIR) signal abnormality; occurrence within the first 3 months after treatment initiation is termed pseudoprogression (PsP) (12–15). These lesions are marked by stability or gradual decrease in signal abnormality over time without additional changes in treatment, and PsP is usually not accompanied by clinical symptoms (3, 12, 13). PsP has been found more frequently in gliomas with hypermethylation of the O-6-methylguanine-DNA methyltransferase (MGMT) gene promoter, which is present in up to ~45% of GBM (3, 14, 16). Presence of the hypermethylated MGMT gene promoter, and thus more frequent occurrence of PsP, is associated with an improved prognosis with proposed mechanism of increased tumor sensitivity to the alkylating effects of TMZ (16). Since the introduction of adjuvant chemotherapy to radiotherapy, the incidence of enhancing lesions in the immediate post-treatment setting has increased, and these lesions are notoriously difficult to distinguish from early tumor progression on conventional magnetic resonance imaging (MRI) (3, 14). Pseudoresponse (PsR) may be seen after treatment with antiangiogenic agents and refers to the transient decrease in enhancement of a treated lesion, which initially mimics treatment response (7, 15). Anti-angiogenic agents such as BVZ and cediranib result in pruning of blood vessels, reduction of permeability of the blood-brain barrier (BBB), and decrease in brain edema, which simulates improvement in enhancement. However, careful scrutiny of the lesion reveals persistent peritumoral T2/FLAIR and diffusion-weighted imaging signal abnormality which worsens on follow-up imaging (as seen in Figure 1), which is related to the progression of infiltrative tumor in the absence of enhancement on conventional MRI (6, 7, 15). Radiation necrosis is part of the spectrum of delayed radiation effects typically seen 6–12 months after the completion of CRT and is a histologic diagnosis (13, 17). The mechanism of tissue damage after radiation therapy (RT) either results from direct axonal injury or secondary white matter injury in the setting of vascular compromise (13, 17, 18). Radiation-induced necrosis is generally progressive and irreversible and may be indistinguishable from PsP or tumor progression *via* conventional MR imaging (19).

MacDonald et al. introduced the traditional criteria for assessment of tumor progression in 1990, which relies on measurement of cross-sectional enhancing diameters as the primary determinant of tumor size (20). However, the limitations of the MacDonald criteria have become apparent over time and include the failure to account for PsP, the lack of definitions of measurable vs. non-measurable disease, and the lack of guidance regarding assessment of non-enhancing tumor and PsR (15, 21). In 2010, the Response Assessment in Neuro-Oncology (RANO) criteria was published to remedy the limitations of the MacDonald with updates addressing PsP, PsR, and non-enhancing tumor progression as well as accounting for the effects of concurrent treatment with corticosteroids (15, 21). Despite these newer guidelines, the challenges of the RANO criteria remain with regards to the lack of standardized grading of the degree of non-enhancing T2/FLAIR perilesional signal which can lead to a high inter-reader discordance rate in radiographic interpretation as well as the limitation of PsP to within 3 months after CRT (14, 15). In actuality, PsP can occur beyond the 3 month cutoff time frame as outlined in the RANO criteria, and cases of early disease progression may be missed using the 3-month cutoff (14, 15, 21). Notable aspects of a modified version of the RANO criteria, proposed by Ellingson et al. in 2017, include the use of contrast-enhanced T1-weighted (ceT1W) subtraction maps which increase lesion conspicuity for a volumetric evaluation of response, the removal of qualitative assessment of non-enhancing tumor, and the use of the initial post-RT time point as the baseline imaging study for evaluating response in newly diagnosed GBM (22).

In clinical practice, a standard classification system called Brain Tumor Reporting and Data System (BT-RADS) for the categorization of post-treatment brain tumor findings was developed to provide greater clarity and improve inter-reader agreement in interpretation and increase the value of the report to ordering clinicians (23). BT-RADS categorizes findings *via* a scoring system ranging from 0 to 4, with 0 indicating baseline findings, 1 indicating improving imaging findings, 2 indicating lack of change, 3 reflecting worsening imaging findings subdivided into three subtypes (3a, resulting from treatment effect; 3b, resulting from an indeterminate combination of tumor progression and treatment effect; and 3c, favoring tumor progression), and 4 indicating findings highly suspicious for tumor progression (23). The use of BT-RADS has been shown to reduce inconsistency and confusion in reporting while improving conciseness (24). In a study of 211 reports, Zhang et al. noted that the use of BT-RADS resulted in more frequent mentioning of important history words such as BVZ use and MGMT status with reduced usage of hedge words as well as reduced length of the total report and impression (24). Gore et al. noted an improvement in clinicians' perceptions of radiology report consistency with decreased ambiguity and increased confidence in reports which use BT-RADS (25).

Despite significant advances in the neuroimaging of gliomas over the last couple of decades, accurate determination of disease progression vs. treatment response is still particularly challenging. When PsP is misinterpreted as treatment failure, appropriate adjuvant therapy may be discontinued prematurely, and the patient may undergo unnecessary invasive repeat biopsy or surgical resection. Conversely, the inclusion of patients with PsP in studies would result in a falsely inflated radiographic response rate (15). Thus, the distinction between recurrence, PsP, and PsR is crucial in determining the most appropriate course of therapy. This review provides a comprehensive overview of the conventional and advanced imaging techniques available in the differentiation of post-treatment changes of gliomas from recurrent disease, with focus on the state-of-the-art in advanced imaging and analytical techniques such as fractional tumor burden (FTB). A brief introduction to techniques in radiomics and artificial intelligence (AI) is also offered. A series of representative cases is provided to illustrate the synthesis of conventional MR and advanced imaging techniques employed in the characterization of the post-treatment appearance of gliomas.

## Conventional MR Imaging

For decades, conventional MRI has formed the cornerstone of post-treatment glioma imaging and provides anatomic characterization, but its diagnostic performance has been hampered by low specificity despite excellent spatial resolution (26, 27). The conventional MR protocol for post-treatment glioma imaging includes the standard T2, FLAIR, pre-gadolinium T1, and post-gadolinium T1 spin-echo sequences and are preferably performed in at least 2 orthogonal planes or acquired with a 3-dimensional (3D) sequence which is reformatted into orthogonal planes (12, 16). These structural sequences provide anatomic lesion localization and evaluate mass effect on the surrounding parenchyma, the ventricular system, and vasculature (12, 28). The physiologic basis for gadolinium contrast enhancement is disruption of the BBB, which reflects tumor infiltration and angiogenesis in gliomas, but alternatively, may result from a variety of factors such as acute reactive changes after surgery or CRT, corticosteroids, and radiation necrosis (12, 17, 29, 30). In a retrospective review of 321 GBM patients undergoing CRT, Young et al. examined a total of 11 MRI signs identified as potentially useful for differentiation between PsP and early progression (EP) but found subependymal enhancement as the only sign to be predictive for EP with a sensitivity of 38%, specificity of 93%, positive predictive value (PPV) of 92%, and negative predictive value (NPV) of 42% (30). They noted that the low sensitivity and NPV suggest the limited utility of this sign in screening for PsP; no sign was found to have a high NPV for PsP which would be most useful clinically (30). In a retrospective review of 27 GBM patients, Mullins et al. found that although individual signs on MRI were not useful predictors for recurrence, a combination of different enhancement patterns may be helpful (31). Specifically, a combination of two signs of corpus callosum involvement and multiple enhancing lesions as well as a combination of the three signs of either corpus callosum enhancement, crossing of the midline, and multiple enhancing lesions or corpus callosum involvement, subependymal spread, and multiple enhancing lesions were significant in favoring recurrence (31).

Although ceT1W images have traditionally been used to define the area of tumor involvement, enhancement is not a sufficient or necessary condition for residual or recurrent disease (16). Conventional MR cannot reliably distinguish treatment effect from recurrence as both involve BBB disruption that result in abnormal gadolinium contrast enhancement (12, 17, 27). Following treatment with anti-angiogenic agents, GBM is more likely to demonstrate an infiltrative pattern of progression, which manifests as increase in non-enhancing peritumoral signal abnormality on T2-weighted and FLAIR sequences (6, 32). An example of decreased enhancement with persistent FLAIR hyperintensity and eventual disease progression is illustrated in Figure 1. Although peritumoral T2 signal may reflect infiltrative tumor or vasogenic edema, classically non-enhancing tumor is characterized by mass effect and architectural distortion such as blurring of the gray-white matter interface and cortical thickening while edema is mostly confined to the white matter (6, 33). In a retrospective study of 26 patients with recurrent gliomas post-BVZ treatment, Schaub et al. found that although an increase in non-enhancing FLAIR signal post-BVZ met the RANO criteria for progression, this finding was not correlated with OS (32). Thus, they recommended that a decision to discontinue BVZ should not be made based on FLAIR-only progression (32). Hattingen et al. demonstrated markedly increased non-enhancing tumor progression using a quantitative T2 relaxation map technique in patients who received BVZ and noted that the degree of change in T2 relaxation time may be an early indicator of OS (34). In a longitudinal study to distinguish abnormal T2 signal as vasogenic edema or infiltrative tumor, Artzi et al. classified the non-enhancing FLAIR lesion area into the two distinct categories of vasogenic edema, as characterized by increased FLAIR signal but decreased perfusion, vs. infiltrative tumor, as characterized by increased perfusion (6). They reported reductions in the enhancing T1-weighted and FLAIR hyperintense lesion volume in the first few weeks after initiation of BVZ indicating a shift to an infiltrative tumor pattern, which was detected via an increase in the volume of the infiltrative tumor category (6). By their classification scheme, the increased percentages of infiltrative tumor at weeks 8 and 16 correlated with PFS (6).

Numerous sources confirm the relatively lower specificity of conventional MRI with respect to advanced imaging techniques. In a meta-analysis examining the diagnostic accuracy of anatomical MR compared to MRI perfusion techniques, van Dijken et al. noted the pooled sensitivity and specificity of conventional MR as 68 and 77%, respectively (26). A systematic review of 17 articles by Shah et al. reported a sensitivity of 89% and specificity of 33% of conventional MR alone (27). Nevertheless, more recent studies using quantitative T1 and T2 mapping and image processing methods have shown potential for the improvement of conventional MRI interpretation. Ellingson et al. used ceT1W subtraction maps to improve the visualization and quantification of tumor volume after BVZ treatment in 160 GBM patients and showed that ceT1W subtraction maps significant improved prediction of PFS at 6 months and OS at 12 months when compared to conventional segmentation methods (35). In a follow-up investigation, Ellingson et al. quantified the volumetric response rate using ceT1W subtraction maps and showed an association between baseline tumor volume and OS as well as a linear correlation between the initial change in tumor volume and OS (36). Ellingson et al. also used T2 maps to quantify the non-enhancing tumor burden with a sensitivity of over 90% and specificity of over 65% (37). Lescher et al. demonstrated that earlier detection of recurrence is achievable with quantitative T1 and T2 relaxation mapping before changes on conventional contrast-enhanced MRI (ceMR) were observed (38). As an example of the potential of machine learning with conventional MRI, Velazquez et al. reported that auto-segmentation of enhancing tumor volumes showed moderate to high agreement with manual segmentation of the same lesions by radiologists and thus offers the potential for reducing inter-reader variability (39).

## Diffusion-Weighted Imaging

### Diffusion-Weighted Imaging and Apparent Diffusion Coefficient

Although diffusion-weighted MRI imaging (DWI) is sometimes considered separately from conventional ceMR, DWI is always acquired as part of the ceMR for brain tumor imaging in clinical practice (40). DWI uses measurements of random water motion to characterize the microstructure of different tissues and is an indicator of cellularity (12, 41, 42). The apparent diffusion coefficient (ADC) is a parameter derived from DWI, which has been reported to have lower values in higher-grade gliomas (12, 43). Tumor cellularity is inversely correlated with ADC value; decreased ADC values can be seen with processes that degrade cellular integrity such as therapy-induced necrosis or tumor growth (41, 44, 45). ADC has been reported to have the highest values in cystic necrosis, followed by vasogenic edema, and has the lowest values in enhancing tumor (45). Some studies have reported ADC values to be higher in the peritumoral vasogenic edema as opposed to infiltrative peritumoral T2/FLAIR signal, although this has not been a consistent finding across studies (17, 46).

An early prospective study of 17 patients by Asao et al. found significantly lower maximum ADC values in patients who developed tumor recurrence compared to those who had radiation necrosis (45). In a retrospective review of 18 GBM patients who developed enhancing lesions after CRT, Hein et al. found that recurrence and treatment effect could be differentiated with mean ADC values and ADC ratios (ratio of ADC value of the enhancing lesion to the ADC value of the contralateral white matter) with lower ADC and ADC ratios significantly associated with recurrence rather than with treatment effect (41). In a study with MR spectroscopy (MRS) and DWI, Zeng et al. confirmed that ADC values and ADC ratios were significantly higher in radiation injury than in recurrent tumor in 55 patients with high-grade gliomas (HGG) (47). Ellingson et al. have shown that histogram or voxel-based analysis of whole-tumor ADC has a sensitivity of 71% and specificity of 69% and can provide early prognostic info in the form of PFS (48). Gupta et al. conducted a retrospective review of 208 patients which demonstrated that 85% of patients with diffusion restricting lesions developed enhancing tumor at the same site 3 months later, suggesting that low-ADC lesions may precede the development of enhancing tumor (49). In a postmortem study with histopathologic correlation, Nguyen et al. determined the optimal ADC threshold for differentiation of hypercellularity and necrosis as 0.736 × 10^−3^ mm^2^/s (50).

Tumor heterogeneity in the post-treatment setting complicates the interpretation of ADC values since lesions are generally an admixture of residual/recurrent tumor and necrotic tissue. Several studies showed equivocal results in the diagnostic performance of DWI and ADC in the post-treatment setting (45, 51). Additionally, in a more recent multiparametric investigation by Liu et al., ADC values did not show significant differences between the recurrence and treatment effect groups (52). Another multiparametric 3-Tesla (T) MR approach by Di Costanzo et al. showed that ADC values were higher but not significantly different in radiation injury than recurrent glioma. Prah et al. sought to spatially discriminate between tumor and treatment effect within contrast-enhancing lesions at different stages of treatment and concluded that ADC could not significantly distinguish treatment effect from recurrence whereas perfusion MR parameters of relative cerebral blood volume (rCBV) and normalized cerebral blood flow (nCBF) were able to (53).

The interpretation of DWI and ADC images should be performed alongside non-contrast T1-weighted images and preferably T2^*^ gradient-echo sequences (GRE) or susceptibility-weighted imaging (SWI) images to avoid false positives in diffusion restricting lesions related to hemorrhage, necrosis, or post-operative tissue injury (54). Smith et al. conducted a prospective study of 44 patients and found that areas of diffusion restriction adjacent to the resection cavity in the immediate post-operative period may resolve and demonstrate subsequent enhancement with eventual encephalomalacia; consequently, they recommend that new enhancement in the immediate post-operative period be interpreted alongside DWI/ADC (55). Mong et al. evaluated persistent diffusion-restricting lesions in patients undergoing BVZ treatment and found that stable diffusion-restricting lesions over time are, in fact, associated with improved outcomes (56).

Although single-shot echo-planar imaging (EPI) is considered the most widely utilized DWI acquisition technique given its advantages of fast acquisition time and availability on clinical MRI scanners, EPI remains sensitive to magnetic field inhomogeneities, resulting in geometric distortions, gradient-induced eddy currents, and T2^*^-induced blurring, especially at high spatial resolution and high *b*-values (57–59). These issues often result from differences in susceptibility between air or venous blood and tissues, as is common in the post-operative setting. Prior solutions have involved using navigator echoes and self-navigated sequences such as periodically rotated overlapping parallel lines with enhanced reconstruction (PROPELLER) and short-axis propeller EPI (SAP-EPI), which reduce sensitivity to magnetic field inhomogeneity and susceptibility artifacts, although these sequences result in increased scan time and thus limited efficiency (59–61). The PROPELLER sequence also corrects for motion degradation as data for each blade can be analyzed to adjust for motion from the patient by oversampling the central k-space with radial sampling resulting in an average of remaining errors (62). Several studies have also demonstrated the advantage of turbo spin echo DWI (TSE-DWI) and readout-segmented echo planar imaging (rsEPI-DWI) in the pediatric brain and at higher magnetic field strengths (58, 62). More recently, Merrem et al. proposed a DWI method which combines a DW spin-echo module with a single-shot stimulated echo acquisition mode MRI (STEAM) sequence and showed that this technique with a modified acquisition and reconstruction strategy results in adequate signal-to-noise ratio (SNR) without susceptibility artifacts and was achievable within 1.5–3 min (57).

In a meta-analysis of 214 patients over 6 studies, Yu et al. noted a pooled sensitivity of 95% with specificity of 83% and supported the auxiliary role of DWI/ADC in the diagnosis of glioma progression (63). Another meta-analysis of 166 patients by Van Dijken et al. noted a pooled sensitivity of 71% and specificity of 87% of ADC values (26) while Du et al. evaluated 17 studies with a total of 656 patients and reported a combined sensitivity of 82% and specificity of 83% (64).

### Diffusion Tensor Imaging

Diffusion Tensor Imaging (DTI) is an extension of DWI and can detect the Brownian motion of water molecules in more than 6 directions (65, 66). DTI is not only used for delineation of white matter tracts for pre-operative guidance but also for assessing the histologic characteristics of tissues and tissue microarchitecture (12, 67). The most common DTI parameters assessed include fractional anisotropy (FA), a measure of the directionality of molecular motion, and mean diffusivity (MD), a measure of the magnitude of diffusion anisotropy (65, 66). Studies have suggested that analysis of FA and ADC values can be used to predict tumor cell density within the core of the tumor or the extent of tumor cell infiltration into the white matter (10, 67, 68). Specifically, the white matter disorganization with tumor infiltration may lead to a decreased FA and increased MD (65). Examples of additional DTI parameters of interest include the axial diffusivity (AD) and radial diffusivity (RD), which refer to the diffusion rate of water parallel and perpendicular to the axon tract, respectively (65).

An early study of 40 patients with intracranial neoplasms including gliomas showed that peritumoral MD and FA values between HGG and low-grade gliomas (LGG) did not differ, but the tumor infiltration index (TII), the difference between expected FA if peritumoral signal were not infiltrated with tumor and the actual FA observed, was significantly different between these two groups (66). However, Kinoshita et al. found that despite the inability of TII to discriminate between vasogenic edema and infiltrative tumor signal, TII correlated with the area of ^11^C-methionine PET uptake, suggesting its usefulness in delineating the extent of tumor cell invasion into the white matter (67). More recently, a retrospective study of 70 patients with GBM conducted by Bette et al. found that the FA within non-enhancing peritumoral areas were significantly lower in regions that eventually developed tumor recurrence, suggesting that recurrence in the non-enhancing peritumoral region might be predictable by FA metrics at baseline (69). Min et al. found that the regression coefficient of RD to AD (RC_RD−AD_) was more effective than FA or TII in distinguishing vasogenic edema from tumor infiltration; when a threshold of 0.6 for RC_RD−AD_ was set, the sensitivity and specificity of the RC_RD−AD_ were 85% and 69% (65). They also corroborated the finding that FA of tumor-infiltrated edema was significantly lower than that of pure vasogenic edema although the FA values of the two types of edema had a wide range of overlap (65). In a retrospective study of 41 patients with enhancing lesions within 6 months after chemoradiation, Wang et al. evaluated several DTI and dynamic susceptibility contrast (DSC) parameters, concluding that the best model to distinguish progression from treatment effect employed a combination of the FA, the linear anisotropy coefficient, and the maximum relative cerebral blood volume (rCBV max). DTI is not without its limitations. For example, crossing or coalescing fibers may lead to false negative images (16). Furthermore, cellular infiltration may lead to more diffusion restriction and possibly failure to detect the tract; edema may also artificially increase diffusion and lead to false positives (16).

## Perfusion-Weighted Imaging

Perfusion-weighted imaging (PWI) includes imaging techniques for measuring brain tumor vascularity and indirectly reveals info on tumor hemodynamics and alterations in capillary permeability (12, 17, 70). As angiogenesis and neovascularization are crucial aspects of glioma tumor biology, PWI has become one of the most commonly used advanced MR imaging techniques used to overcome the non-specific features of conventional MRI (8, 71, 72). Variations in regional blood flow and volume reflect changes in tumor vascularity over the course of treatment (2, 16, 72). The most commonly employed PWI techniques include DSC, dynamic contrast-enhanced MRI (DCE), and arterial spin labeling (ASL) MRI (12, 17). Evidence in the literature shows that selection of either the DSC or the DCE technique does not affect overall diagnostic performance in distinguishing between recurrence and treatment effect (73).

### Dynamic Susceptibility Contrast MRI

DSC perfusion imaging measures the susceptibility-induced T2^*^ signal loss from the injected contrast bolus as it passes through the capillary bed, with the loss of signal depicted as a signal intensity-time curve (12, 17, 74). Subsequently, the AUC is used to derive the relative cerebral blood volume (rCBV), which has become an imaging biomarker for angiogenesis and is widely applied in the diagnosis and treatment monitoring of brain tumors (12, 17, 74). Cerebral blood flow (CBF) can also be easily calculated from the contrast-concentration over time curve (12, 17, 74). rCBV has been used to distinguish higher grade from lower grade gliomas and has been found to be elevated in the peritumoral T2/FLAIR signal of infiltrative glioma as compared to peritumoral vasogenic edema (6, 10, 75). In the post-treatment setting for gliomas, rCBV has been found to be elevated in recurrent or residual tumor more than in PsP or radiation necrosis (50, 76).

Early on, Sugahara et al. demonstrated that normalized rCBV ratios could be used to classify enhancing lesions in treated brain tumors as recurrence or non-neoplastic enhancing tissue, which they confirmed with thallous chloride single-photon emission tomography (^201^Tl-SPECT) (77). Specifically, they noted lesions having a rCBV ratio >2.6 as more likely to be recurrence and lesions with a rCBV ratio <0.6 as more likely to reflect treatment-related changes (77). In a retrospective analysis of 57 post-radiotherapy GBM patients, Barajas et al. found that the rCBV as well as the mean, maximum, and minimum peak height (PH) were significantly higher in patients with recurrent GBM than in patients who had radiation necrosis (78). Boxerman et al. investigated a subset of patients with HGG on a phase 2 clinical trial with TMZ and noted that the change in rCBV at first follow-up visit as well as the overall trend in rCBV rather than the mean rCBV at baseline was significantly different between PsP and progressive disease (79). Mangla et al. also found that the percent change in rCBV at 1 month after CRT with TMZ correlated with survival (80). Blasel et al. found that the maximum rCBV better differentiates tumor progression from recurrence rather than the mean rCBV; they noted that a maximum rCBV of 2.6 had a 78% sensitivity and 86% specificity to detect tumor progression but was not predictive for OS (81). Several studies investigated the diagnostic advantage and clinical value of adding PWI to conventional MR imaging and DWI. Geer et al. reported that with the addition of PWI, confidence in assessment of the post-treatment status increased in 40% of neuroradiologists and 56% of clinicians (82). Kim et al. noted that the addition of DSC (as well as DCE) significantly increased the area under the receiver operating characteristic curve (AUC) for two readers' prediction of recurrent disease (73).

Several larger-scale studies have also shed light on the diagnostic performance of DSC MR in distinguishing recurrence from treatment effect. Snelling et al. reviewed 337 scans from 64 patients and noted the sensitivity and specificity of PWI for HGG as 61 and 88% and for LGG as 86 and 89%, respectively. A meta-analysis including 28 articles by Patel et al. reported a pooled sensitivity of 90% and specificity of 89% for DSC (83). A systematic review and meta-analysis by Wang et al. noted a pooled sensitivity and specificity of 83 and 83% for DSC, respectively (84). In corroboration of these results, a systematic review and meta-analysis by Van Dijken et al. noted a pooled sensitivity and specificity of 87 and 86% for DSC, respectively (26).

One of the disadvantages of DSC is that quantitative rCBV measurements are not feasible given the poor reliability of arterial input functions and unknown voxel-wise contrast agent T2^*^ relaxivity (85). However, the normalization of rCBV values can provide a means for semiquantification against each patient's own tissue for internal reference. The convention of a user-defined region-of-interest (ROI) within the contralateral normal-appearing parenchyma is susceptible to significant variability in rCBV measurements (85). The use of standardized intensity scales for rCBV maps without the need for user-defined reference ROIs has shown potential for improving the consistency of rCBV measurements (85). Additional limitations of DSC include sensitivity to magnetic field inhomogeneities and to the presence of vascular injury in the post-treatment setting; for example, telangiectasias and aneurysms may increase rCBV while radiotherapy-induced microbleeding in recurrence may result in decreased rCBV (86, 87). A major pitfall of DSC is its underlying assumption that the BBB is intact without contrast leakage or recirculation; if contrast leakage is present, then overshooting may be seen on the DSC time curve (17, 88, 89). Traditionally, a preload bolus technique is used to overcome this, which can be used by itself or in conjunction with DCE (90). For example, a standardized PWI-MR imaging protocol investigated by Anzalone et al. involved splitting a full dose of gadolinium (10 mL gabobutrol)—an initial half-dose (5 mL) bolus was injected 50 s after the start of DCE acquisition and a second half-dose (5 mL) bolus was injected 16 seconds after the start of DSC acquisition (91). Using a full dose for each bolus provides for a higher contrast-to-noise ratio, although a half dose for each bolus with the use of a low flip angle (30 degrees) may provide a good alternative with only a modest decrease in accuracy (90). The preload contrast administration serves to reduce the contaminating T1 effects before the bolus and may also reduce the concentration gradient of contrast extravasation; preload administration is especially recommended for single-echo DSC-MR with high flip angles (91, 92). However, multi-echo instead of single-echo acquisitions and the use of a low flip angle without a preload dose have been proposed for leakage correction (72, 93). Additionally, variation in post-processing methods and software modeling result in inconsistencies in rCBV calculation (88, 94). A recently published set of consensus recommendations for a DSC protocol in HGG by Boxerman et al. is a step toward increased standardization of the DSC technique in post-treatment glioma imaging (90).

More recently, voxel-based, semiquantitative, and quantitative approaches including machine learning techniques have been employed in efforts to achieve a more sensitive and accurate analysis of rCBV. Tsien et al. created parametric response maps (PRM) using DSC parameters such as rCBV to quantify early hemodynamic alterations during treatment and noted a significant difference between the PRM of patients with PsP and those with disease progression (29). In a retrospective study using a semiquantitative histogram analysis of parameters derived from the normalized CBV, Kim et al. showed that the peak height position (PHP) was an independent predictor for the differentiation of recurrence and post-treatment changes with a sensitivity of 90% and specificity of 91% at an optimum threshold of 1.7 while the maximum value (MV) showed a sensitivity of 96.5% and specificity of 93% with an optimum threshold of 2.6 (95). Sanders et al. recently conducted a comparison of synthetically generated DSC-derived rCBV maps from DCE MRI and compared them to the real rCBV maps generated from the acquired DSC MRI and noted a strong correlation between the synthetic and real rCBV maps (74). Thus, the ability to reliably generate a rCBV map from DCE data could potentially spare the patient an additional bolus of gadolinium intravenous (IV) contrast (74).

### Role of Fractional Tumor Burden

Gliomas, particularly GBM, are characterized by marked tumor heterogeneity, with areas of solid tumor mixed with necrosis and peritumoral edematous brain parenchyma which is infiltrated by tumor cells (2, 10). Intratumoral heterogeneity is even more important to recognize in the post-treatment setting where the treated lesion is characterized by an admixture of residual tumor cells and necrosis from the cytotoxic effects of CRT (53, 96). Although PWI has great potential in differentiating recurrent tumor vs. treatment effect, its diagnostic performance is limited by the use of gross regional metrics, whereby the mean, median, or maximum values are derived from the entirety of a lesion with abnormal enhancement or T2/FLAIR hyperintensity (53). Consequently, an accurate characterization of the proportion of a treated lesion that is recurrent or residual tumor vs. treatment effect is limited by spatial averaging, which may account for substantial variation in the thresholds for PWI parameters reported in the literature (53). Additional challenges of using PWI-derived metrics include inter-operator subjectivity in the “hot spot” ROI selection for calculation of rCBV values when a ROI is manually selected in an area of tumor with the highest rCBV on a single image (88). However, this method underestimates the entire volume of tumor as well as fails to account for tumor heterogeneity (97). Theoretically, stereotactic tissue sampling throughout a new or worsening area of enhancement of T2/FLAIR signal abnormality would provide insight into how much of a treated lesion consists of residual or recurrent tumor vs. treatment-induced necrosis (53). However, this approach would be prohibitively invasive and unlikely to be feasible in clinical practice. Regional tumor heterogeneity can also thwart diagnostic confirmation *via* tissue sampling (85). Thus, a non-invasive imaging technique for quantifying tumor heterogeneity has the potential for preventing unnecessary invasive biopsies or repeat resections and more accurately informing clinical decision-making.

The fractional tumor burden (FTB) is an analytical technique that uses PWI data to quantitatively evaluate intralesional heterogeneity in the post-treatment setting. Specifically, FTB is defined as the volumetric fraction of tumor voxels higher than a specified rCBV threshold (97). This method provides perfusion characterization of the entire lesion by using per-voxel measurements of CBV rather than relying on a single value to represent the perfusion of the entire lesion (97). The FTB technique also removes operator dependence with the “hot spot” ROI method which improves reproducibility (72, 97). Another advantage of using FTB is decreased reliance on the magnitude of rCBV values which may be quite variable between institutions secondary to differences in image-acquisition and post-processing techniques (85, 97). Critical to FTB is the use of rCBV thresholds for defining tumor vs. treatment effect.

The groundwork for development of the FTB technique was established by Gasparetto et al. who showed that recurrent tumor and treatment-related necrosis could be discriminated based on whether the rCBV of each lesion was above or below predefined rCBV thresholds (98). In a retrospective analysis of 30 brain tumor patients, they also showed that a unit increase in rCBV increases the probability of recurrence from 57 to 90% and measured an accuracy of 97% in distinguishing enhancing lesions as recurrence or treatment-effect (98). Subsequently, Hu et al. introduced the FTB metric and demonstrated that the FTB of GBM patients with recurrence strongly correlated with histologic tumor fraction as well as OS (96), validating the potential for FTB to serve as an imaging biomarker for tumor progression. They also found that the rCBV mean and mode correlated less strongly with histology compared to FTB and did not correlate with OS (96), which may reflect the advantage of FTB in its ability to account for tumor heterogeneity. Furthermore, Hu et al. noted that a rCBV threshold of 1 differentiates treatment effect and recurrence with 100% accuracy (96). In support of these results, Hoxworth et al. demonstrated that FTB can distinguish between low vs. high histologic tumor content when a set of threshold cutoff values are applied (85). More recently, Iv et al. used preset normalized rCBV thresholds of 1.0 and 1.75 to define low, intermediate, and high FTB in a retrospective evaluation of 47 HGG patients (97). They demonstrated that recurrent tumor exhibited higher FTB and rCBV than treatment effect while treatment effect exhibited lower FTB and rCBV values than recurrence (97). However, intermediate FTB values did not reliably differentiate between recurrence and treatment effect (97). Additionally, there was good consensus agreement among the five physicians involved in this study regarding whether the use of FTB would inform short-term management plans for each case (97). This body of evidence suggests that FTB has the potential to estimate tumor burden accurately and expeditiously during a single MRI study as opposed to obtaining serial ceMR studies for follow-up, which could be used to triage/identify patients who may not need an invasive biopsy or repeat resection.

**Figure 2:**
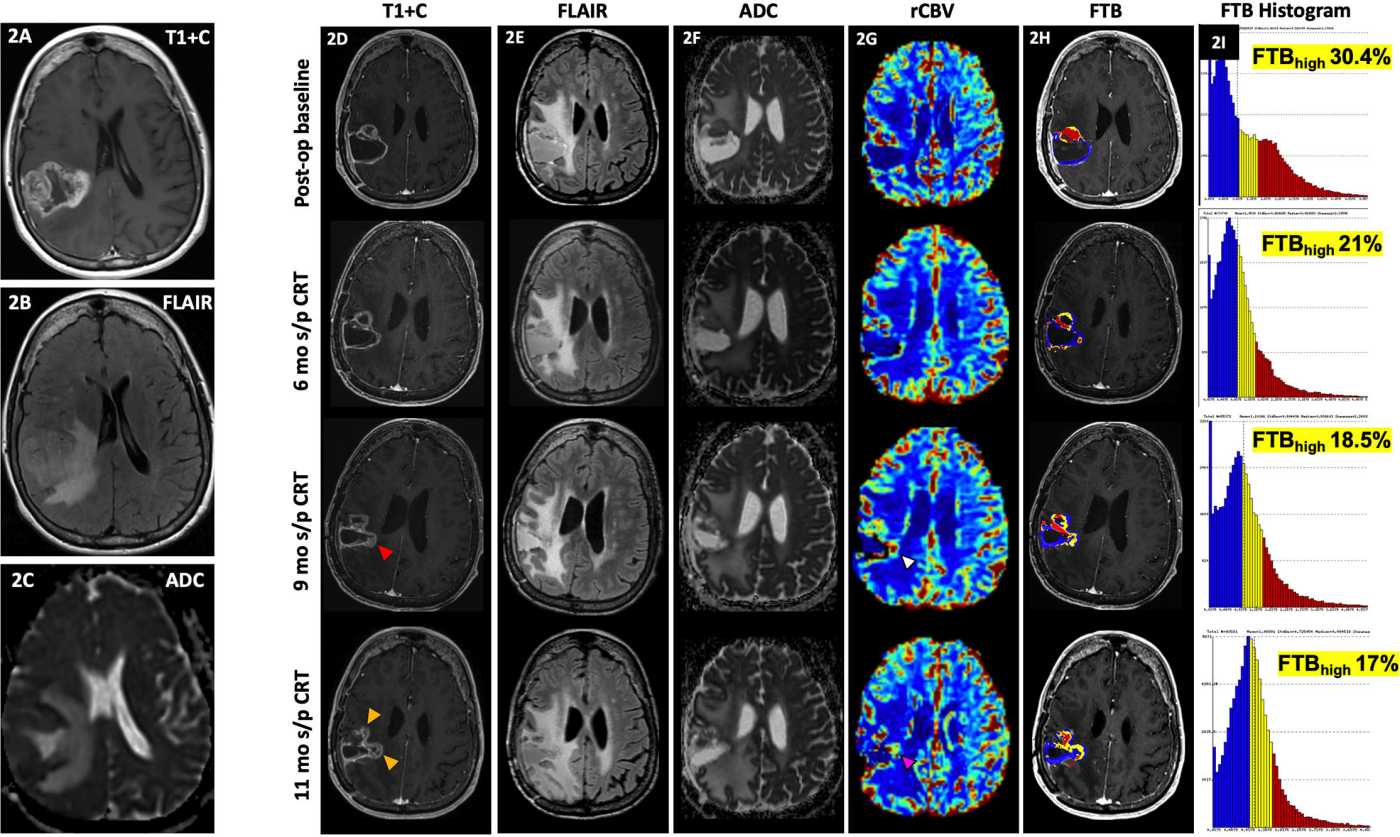
Delayed pseudoprogression. A 71-year-old female with GBM (IDH wt, MGMT+) initially presented with an enhancing right parietal mass **(A)** with extensive locoregional mass effect, perilesional FLAIR hyperintensity **(B)**, and diffusion restriction **(C)**. Her post-op baseline MR showed mild peripheral enhancement of the resection cavity most pronounced at the anterior aspect [**(D)**, top row] with associated perilesional FLAIR hyperintensity [**(E)**, top row], and some diffusion restriction [**(F)**, top row] and increased rCBV [**(G)**, top row]. At 6 mo s/p CRT, there is persistent peripheral enhancement of the resection cavity [**(D)**, 2nd row] with slightly increased FLAIR hyperintensity [**(E)**, 2nd row], less prominent diffusion restriction [**(F)**, top row], and similar to slightly decreased rCBV [**(G)**, 2nd row). At 9 mo s/p CRT, there is mildly increased enhancement at the medial aspect of the resection cavity [**(D)**, 3rd row, red arrowhead) with similar perilesional FLAIR hyperintensity [**(E)**, 3rd row], less prominent diffusion restriction [**(F)**, 3rd row], and slightly increased rCBV [**(G)**, 3rd row, white arrowhead]. At 11 mo s/p CRT, there is mildly increased nodular enhancement at the medial aspect of the resection cavity [**(D)**, 4th row, yellow arrowhead], increased perilesional FLAIR hyperintensity [**(E)**, 4th row], similar to mildly increased diffusion restriction [**(F)**, 4th row], and increased rCBV [**(G)**, 4th row, pink arrowhead]. From her post-op baseline scan to the 11 mo s/p CRT time-point, areas of high fractional tumor burden (FTB_high_), within the lesion as reflected by the red areas within the lesion, visually show gradual decrease over time [**(H)**, in order from top to bottom rows], which is also supported by the progressive quantitative decrease in FTB_high_ percentages over time as shown on the corresponding histograms [**(I)**, in order from top to bottom rows]. Despite mild worsening of conventional MR findings, the gradual decrease in FTB_high_ suggests treatment effect rather than progression. Resection of the lesion was nonetheless performed, and histopathology demonstrated necrosis. Since the worsening of conventional MR findings occurred >3 months after CRT, this is considered delayed Psp or treatment necrosis. ADC, apparent diffusion coefficient; ASL, arterial spin labeling; CRT, chemoradiotherapy; FLAIR, fluid-attenuated inversion recovery; FTB, fractional tumor burden; GBM, glioblastoma; IDH, isocitrate dehydrogenase; MGMT, O-6-methylguanine-DNA methyltransferase; mo, months; op, operative; Psp, pseudoprogression; rCBV, relative cerebral blood volume; s/p, status post; T1+C, post-contrast T1-weighted images; tx, treatment; wt, wildtype.

**Figure 3:**
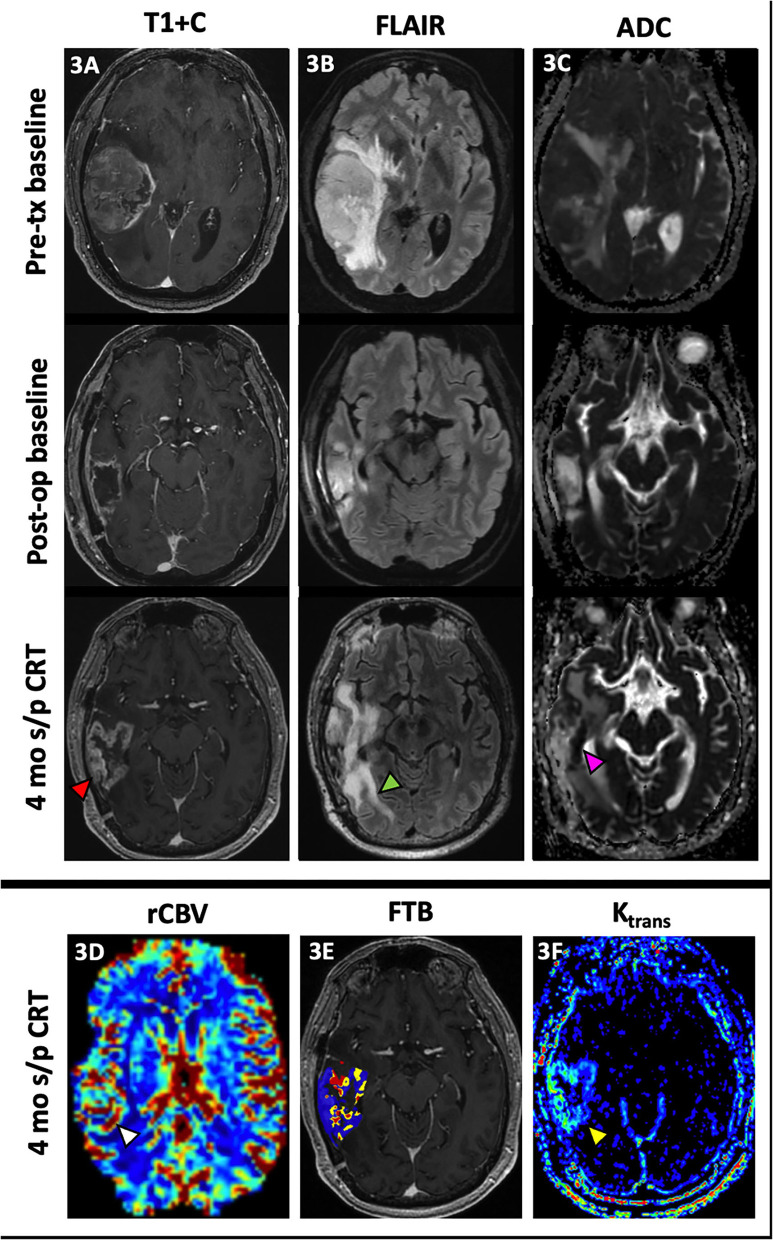
Admixture of treatment effect and tumor. A 68 year-old male with GBM (IDH wt, MGMT+) initially presented with a large enhancing right temporal lobe mass [**(A)**, top row] with extensive perilesional FLAIR hyperintensity [**(B)**, top row] and diffusion restriction [**(C)**, top row]. After resection, there is mild peripheral enhancement of the resection cavity [**(A)**, middle row] with decreased perilesional FLAIR hyperintensity [**(B)**, middle row] and no significant diffusion restriction [**(C)**, middle row]. At 4 mo s/p CRT, new peripheral nodular enhancement of the resection cavity [**(A)**, bottom row, red arrowhead] is noted with interval increase in perilesional FLAIR hyperintensity [**(B)**, bottom row, green arrowhead] and increased diffusion restriction most pronounced at the medial aspect of the resection cavity [**(C)**, bottom row, pink arrowhead]. There is also associated increased rCBV [**(D)**, white arrowhead], a mix of low and high fractional tumor burden (blue and red, respectively) **(E)** and vascular permeability [**(F)**, yellow arrowhead]. This lesion was resected with pathology demonstrating predominantly radiation necrosis (Ki-67 of 5%) but with some neoplastic cells. The heterogeneous nature of this treated lesion is supported by the mix of low and high fractional tumor burden seen on the FTB map. ADC, apparent diffusion coefficient; CRT, chemoradiotherapy; FTB, fractional tumor burden; GBM, glioblastoma; IDH, isocitrate dehydrogenase; K^trans^, volume transfer coefficient; MGMT, O-6-methylguanine-DNA methyltransferase; mo, months; op, operative; rCBV, relative cerebral blood volume; s/p, status post; T1+C, post-contrast T1-weighted images; tx, treatment.

Figure 2 demonstrates how FTB can be useful as a non-invasive method of depicting tumor heterogeneity and determining if an enhancing lesion consists of more residual/recurrent tumor vs. treatment-induced necrosis. Despite mildly worsening imaging features on conventional MR, visual assessment of the FTB map does not demonstrate an increase in areas of high blood volume within the lesion. In support of this, there is downtrending of the FTB_high_ percentages on the FTB histograms over several follow-up studies. The FTB findings supported the pathology result of necrosis from resection in this case. FTB also appropriately depicts tumor heterogeneity in Figure 3 where new enhancement and FLAIR hyperintensity is seen in the resection bed 4 months s/p CRT. Although the rCBV and ktrans (see DCE section below) were both increased, the FTB showed a more heterogenous mix of low and high blood volumes throughout the lesion. Pathology results from resection confirmed the lesion as a mix of tumor cells and radiation necrosis.

### Dynamic Contrast-Enhanced MRI

**Figure 4:**
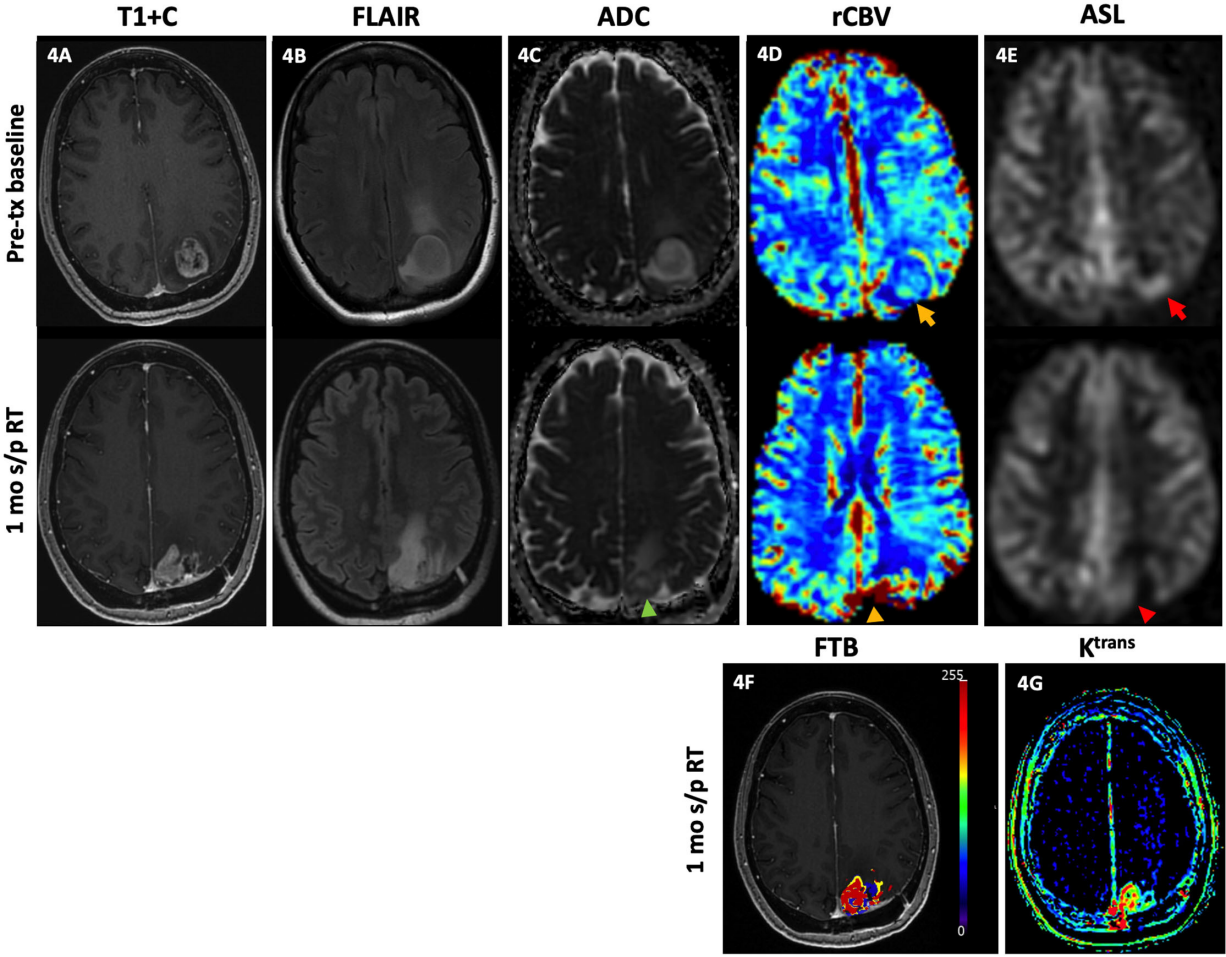
Tumor recurrence. A 49-year-old female with GBM (IDH wt, MGMT+) was initially diagnosed with an enhancing left parietal mass [**(A)**, top row] with associated lesional and perilesional FLAIR hyperintensity [**(B)**, top row], diffusion restriction [**(C)**, top row], increased rCBV [**(D)**, top row, yellow arrow], and ASL hyperperfusion [**(E)**, top row, red arrow]. She then underwent gross total resection and concurrent CRT with minimal residual enhancement of the resection cavity margins without nodular enhancement, diffusion restriction, or increased rCBV or ASL signal (not shown). One month after completion of RT, there is new focal nodular enhancement at the medial aspect of the resection bed [**(A)**, bottom row] with associated FLAIR hyperintensity [**(B)**, bottom row], diffusion restriction [**(C)**, bottom row, green arrowhead], increased rCBV [**(D)**, bottom row, yellow arrowhead], and ASL hyperperfusion [**(E)**, bottom row, red arrowhead]. There is increased FTB_high_, within the lesion as reflected by the red-colored areas **(F)**, and increased vascular permeability on the K^trans^ map **(G)**. Note on the rCBV, FTB, and K^trans^ maps, a color scale of blue to red represents the incremental increase in values from low to high. The findings of increased enhancement, FLAIR hyperintensity, diffusion restriction, rCBV, FTB_high_ and K^trans^ favor recurrence. Subsequent resection of the lesion showed recurrent GBM. ADC, apparent diffusion coefficient; ASL, arterial spin labeling; FLAIR, fluid-attenuated inversion recovery; FTB, fractional tumor burden; GBM, glioblastoma; IDH, isocitrate dehydrogenase; K^trans^, volume transfer coefficient; MGMT, O-6-methylguanine-DNA methyltransferase; mo, months; rCBV, relative cerebral blood volume; RT, radiation therapy; s/p, status post; T1+C, post-contrast T1-weighted images; tx, treatment; wt, wildtype.

DCE perfusion imaging is accomplished by consecutively acquiring a series of T1 weighted images before, during, and after IV gadolinium contrast administration to produce a contrast signal time intensity curve (2, 17). The dynamic T1 signal intensity can then be used to characterize the concentration of contrast between the intravascular and extravascular spaces using model-dependent and model-free parameters (2). The volume transfer coefficient, or K^trans^ is the most commonly calculated parameter and reflects the vascular permeability between the plasma and extracellular space (2, 12, 99). However, K^trans^ more closely reflects vascular permeability when the BBB is intact; when the BBB is disrupted by tumor, K^trans^ is more representative of the CBF (17). Evidence in the literature postulates that K^trans^ is increased in brain tumors likely due to the formation of immature hyperpermeable vessels in the setting of neovascularization; thus, K^trans^ may be a useful imaging biomarker for tumor proliferation/ recurrence (2, 100–102). Examples of increased K^trans^ are shown in Figures 3F, 4G. The volume of extravascular extracellular space per unit of volume of tissue, V_e_, has also been used to characterize brain tumors (100, 101). Over two studies, Jia et al. demonstrated higher values for K^trans^ and V_e_ in HGG than in LGG (100, 101), which reflects increased microvascular permeability of tumor vessels and increased BBB disruption in more aggressive gliomas. Additional studies have shown that K^trans^ and V_e_ correlate with the Ki-67 index, a tumor cell proliferation marker that has been useful for glioma grading and prognosis, as well as VEGF expression in gliomas, further supporting the use of these parameters to characterize neovascularization in gliomas (103, 104). Ahn et al. noted that K^trans^ values were higher in GBM patients with methylated MGMT promoters, which has been associated with PsP and treatment response (105). The V_p_, or plasma volume, derived through pharmacokinetic modeling is also a biomarker of tumor neoangiogenesis (86).

In a retrospective study of 37 GBM patients post-CRT with new or increased enhancing lesions, Thomas et al. found that K^trans^ and V_p_ were lower in PsP than in disease progression and found that a K^trans^ of >3.6 had a sensitivity and specificity of 69% and 79% for disease progression (106). Yun et al. also found a lower mean Ktrans in PsP than in disease progression among 33 GBM patients and noted that the mean Ktrans value could predict PsP with 59% sensitivity and 94% specificity (107). Morabito et al. compared the diagnostic accuracy of DSC and DCE MRI in differentiating recurrence from treatment-induced necrosis and concluded that K^trans^ and rCBV are significantly different between patients with recurrent tumor and radiation necrosis with a sensitivity and specificity of 89 and 97% when an optimal cut-off value of 28.76 for K^trans^ was used (108). In exploration of a different DCE parameter, Chung et al. used a bimodal histogram analysis of the AUC ratio (ratio of the initial AUC to the final AUC) derived from the DCE signal intensity-time curve to distinguish recurrence from radiation effect with a sensitivity of 94% and specificity of 88% (109). Bisdas et al. investigated several DCE parameters in 18 glioma patients undergoing treatment and found that the Ktrans and AUC were significantly different between recurrent tumor lesions and radiation-induced necrosis (110). In their retrospective study investigating the differentiation of infiltrative tumor from vasogenic edema in the post-BVZ setting, Artzi et al. found that the infiltrative tumor regions were characterized by higher K^trans^ and V_p_ than regions of vasogenic edema (6).

More recently, Elshafeey et al. built a machine learning model using radiomic features from K^trans^ and rCBV maps and were able to achieve a sensitivity of 91%, specificity of 88%, and AUC of 89% for differentiation of PsP from disease progression; in particular, the diagnostic performances of models constructed using the Ktrans and rCBV maps separately were equally high (111). Several meta-analyses report the pooled sensitivities and specificities of DCE MRI for distinguishing recurrence from treatment effect as 92 and 85% (26), 89 and 85% (83), and 73 and 80% (84), respectively.

Compared to DSC MRI, DCE MRI offers better spatial resolution and is not affected by susceptibility artifact; DCE technique also allows absolute quantification of perfusion parameters whereas with DSC technique, CBV values must be normalized to the contralateral normal brain parenchyma (12, 86). However, DCE is somewhat more challenging to implement clinically given the need for pharmacokinetic modeling (21).

### Arterial Spin Labeling

Arterial spin labeling (ASL) is a non-contrast MR perfusion technique that uses inversion pulses to magnetically label inflowing arterial blood protons, which then act as natural endogenous tracers for flow rate measurement (17, 112). Thus, ASL is theoretically unaffected by the condition of the BBB, as opposed to conventional ceMR (113, 114). Although ASL imaging does not directly measure the CBV, the CBF can be quantified by comparing the differences in signal between labeled and non-labeled images; studies have shown blood volume and flow are strongly correlated (17, 112, 115). Areas of elevated CBF on ASL images suggest hypervascularity and angiogenesis, which may be predictive of glioma recurrence (17, 116).

Ozsunar et al. compared the diagnostic performance of ASL, DSC, and fluorodeoxyglucose positron emission tomography (FDG-PET) *via* visual and quantitative analysis in 30 glioma patients and found a sensitivity of 88% for ASL which increased to 94% using a normalized cutoff ratio of 1.3, with lower sensitivities of 81 and 86% for DSC and FDG-PET, respectively (117). Choi et al. applied a grading system to the ASL signal and demonstrated ASL grade to serve as an independent predictor in differentiating PsP from early progression with sensitivity of 79% and specificity of 64% (107). More recently, Manning et al. evaluated the ability of ASL and DSC to differentiate recurrence from PsP and noted that the CBF measured *via* ASL technique had the highest AUC and misclassified the least number of cases (118). Pellerin et al. reported that compared to 3,4-dihydroxy-6-^18^F-fluoro-L-phenylalanine (^18^F-DOPA) PET, ASL demonstrated a higher specificity of 100% in tumor isocontour maps whereas ^18^F-DOPA-PET demonstrated a higher sensitivity of 94% (119). In a meta-analysis of 20 studies with a total of 939 patients evaluating the value of PWI in the post-treatment glioma setting, the pooled sensitivity and specificity for ASL were 79 and 78%, respectively, with an AUC of 0.88 (84). An additional meta-analysis and systematic review performed by Liu et al. included 10 studies with a total of 368 patients and found that the CBF, rCBF, and rCBV were higher in patients with glioma recurrence than in patients with treatment effect (114).

The advantages of ASL technique include high signal-to-noise ratio (SNR), avoidance of susceptibility artifacts, immediate availability of images, ease of quantification, and lack of IV contrast administration (71). ASL also has better performance around resection cavities as it is less sensitive to prominent surrounding vessels (112, 119). In contrast to DSC technique, the absolute as opposed to the relative CBF values can be measured, which allows for ease of cross-study comparisons and facilitates longitudinal follow-up studies (118). However, ASL is limited by low spatial resolution, lengthened scan time, and motion degradation (118).

Figure 4 demonstrates elevated rCBV, ASL, increased areas of high fractional tumor burden (FTB_high_), and vascular permeability which correlate with conventional MR findings of increased enhancement and perilesional FLAIR hyperintensity in a GBM patient with pathology-proven recurrence upon resection.

## MR Spectroscopy

MR spectroscopy (MRS) differentiates between metabolites using nuclear magnetic resonance and the excellent spatial localization provided by MRI (120–122). The main metabolites of interest are N-acetylaspartate (NAA), a marker of neuronal viability; creatinine (Cr), a marker for cellular metabolism; and choline (Cho), a marker of cell membrane turnover (12, 120–122). Lipid and lactate peaks can be seen as indicators of necrosis and hypoxia, and myoinositol (MI), a glial marker synthesized primarily in astrocytes, may be elevated in LGG (12, 120–122). Ratios of Cho/NAA and Cho/Cr are often used to express increased Cho levels, indicating increased cell density (120). The MRS profile of gliomas has been widely investigated and is recognized as an increase in Cho with decreases in NAA, Cr, and MI (121, 122), as demonstrated in Figures 1L,M. In the setting of radiation necrosis, Cho and Cr are reduced while lipids and lactate levels may be elevated (120, 122). Mapping of Cho levels has been suggested as a method for defining tumor boundaries (120). However, there is significant overlap in the MRS findings of a recurrent lesion and RT-related changes given the degree of tumor heterogeneity (120). Although MRS has been useful in the differential diagnosis and histologic grading, its use in differentiating glioma recurrence from treatment effect is more effective when combined with other advanced imaging techniques (120, 122).

MRS may detect metabolic changes in recurrent tumor before a significant change is seen in the volume of enhancement (122, 123). Artzi et al. verified their PWI-based classifications of lesions as infiltrative tumor or vasogenic edema using MRS and found significantly higher Cho/Cr for the infiltrative tumor category of lesions (6). In areas of infiltrative tumor, confirmatory findings on MRS can extend beyond the area of enhancement and/or T2/FLAIR signal abnormality on conventional MR (120). Steidl et al. noted substantially increased MI concentrations in tumor and control tissue during BVZ treatment; higher MI concentrations at baseline in the control tissue and higher differences between the control and tumor tissue correlated with longer survival (124). Results from some studies suggest a more limited role of MRS in differentiating recurrence from treatment effects. Rock et al. noted that MRS cannot reliably differentiate between a mix of residual/recurrent tumor and radiation necrosis, although it is able to distinguish pure necrosis from tumor (125). More recently, a multiparametric evaluation at 3T by Liu et al. did not demonstrate any significant difference between the Cho/Cr, Cho/NAA, and NAA/Cr of recurrent lesions vs. treatment effect.

Nevertheless, van Dijken et al. report a pooled sensitivity and specificity of 91% and 95% for MRS in a systematic review and meta-analysis of 203 patients in 9 studies (26). Another systematic review and meta-analysis of 455 patients from 18 articles by Zhang et al. reported a pooled sensitivity and specificity of 83% and 83% with AUC of 0.9 for Cho/Cr and 88% and 86% with AUC of 0.9 for Cho/NAA (126). Given the moderate diagnostic performance of MRS, they recommended that MRS should be used in conjunction with additional advanced imaging techniques in the imaging evaluation of post-treatment gliomas (126).

The challenges of employing MRS in the post-treatment evaluation of gliomas include its low spatial resolution for small lesions; non-standardization of equipment, pulse sequences, and post-processing methods; as well as variability in results due to manually selected ROIs and lesion heterogeneity (17, 122, 127). Specifically, the marked tumor heterogeneity that defines treated gliomas significantly influences the metabolic spectrum of the sample depending on the ROI chosen within the lesion. In the case of overlapping findings between recurrence and treatment effect, such as the elevation in Cho after CRT due to cell injury and astrogliosis or the decrease in NAA seen with neuronal damage both in tumor and necrosis, Weinberg et al. suggest a subsequent MRS evaluation in 6–8 weeks to follow the trend in Cho levels, which should normalize over time in the case of treatment effect (122). Conversely, the advantages of MRS consist of not requiring IV contrast administration and potential greater accessibility than metabolic imaging as a problem-solving tool in the imaging of post-treatment gliomas.

## Metabolic/Functional Imaging

### SPECT

Single photon emission computed tomography (SPECT) refers to the use of radiopharmaceuticals that localize to areas of tumor with gamma camera imaging. The most common SPECT radiopharmaceuticals used in post-treatment glioma imaging have been ^201^Tl and technetium-99m sestamibi (^99m^Tc-MIBI) although other less commonly available radiopharmaceuticals such as Tc-99m dimercaptosuccinic acid (^99m^Tc-DSMA) and Tc-99m glucoheptonate (^99m^Tc-GHA) have also been employed. The advantages of performing SPECT imaging include the low cost, wider availability, and ease of interpretation, especially with the aid of an accompanying low-dose computed tomography (CT) (128).

^201^Tl is a potassium analog and undergoes active transport through the adenosine triphosphate (ATP) cell membrane transporter; thus, ^201^Tl uptake is related to cellular growth with non-viable tissue exhibiting little ^201^Tl uptake (40, 129). ^99m^Tc-MIBI passively diffuses through the cell membrane under the transmembrane potential with higher uptake in malignant cells, but its physiological biodistribution in the choroid plexus as well as the temporalis and extraocular muscles limits its use in brain tumor imaging (130, 131). ^99m^Tc-DSMA is thought to be a phosphorus anion (${\text{PO}}_{4}^{3-}$) analog with its intracellular accumulation linked to phosphate uptake and kinase pathway activation; its advantage over ^99m^Tc-MIBI is a lack of uptake across the intact BBB (128). Thus, ^99m^Tc-MIBI is not taken up in normal brain tissue or choroid plexus (128). ^99m^Tc glucoheptonate (GHA) is a BBB agent and a structural glucose analog with increased uptake in tumor cells (132). ^99m^Tc radiopharmaceuticals are generally favored over ^201^Tl currently due to the higher spatial resolution and lower radiation dose achievable with ^99m^Tc rather than ^201^Tl (40, 128).

In a retrospective review of 19 patients, Tie et al. reported the sensitivity and specificity of ^201^Tl-SPECT for diagnosing recurrence to be 83 and 100%, respectively (129). They observed that the ^201^Tl-SPECT result correctly determined management in 29% and aided the management in 48% of patients and found the diagnostic accuracy of ^201^Tl-SPECT to be superior to conventional MR (129). A study of 201 patients, Le Jeune et al. compared ^99m^Tc-MIBI-SPECT uptake to stereotactic biopsy results in treated glioma patients and reported the sensitivity, specificity, and accuracy for detecting tumor recurrence as 90, 91.5, and 90.5%, respectively (130). They also observed that a ^99m^Tc-MIBI-SPECT diagnosis of anaplastic degeneration of LGG was sometimes seen earlier than with conventional MR or clinical characteristics (130). More recently, Roshdy et al. evaluated 30 HGG patients with clinical or radiologic suspicion for recurrence *via* ^99m^Tc-MIBI-SPECT/CT and noted that patients with MIBI uptake positive for recurrence were associated with poor survival (131). Amin et al. compared ^99m^Tc-DSMA-SPECT with MRS for the detection of residual or recurrent disease in 24 glioma patients and found the sensitivity and accuracy for SPECT to be 89 and 92% vs. 61 and 71% for MRS, respectively (128). Santra et al. found the sensitivity, specificity, and accuracy of ^99m^Tc-GHA-SPECT to be 87%, 97%, and 89% compared to 95%, 24%, and 71% for ceMR, with ^99m^Tc-GHA-SPECT demonstrating substantially higher specificity and accuracy than ceMR (132). In a recent study, Rani et al. compared the diagnostic performance of ^99m^Tc-bismethionine-DTPA (MDM) SPECT/CT with DSC MRI for the detection of recurrence and noted a comparable sensitivity and specificity between the two imaging modalities, i.e., 92 and 79% for ^99m^Tc-MDM SPECT/CT vs. 92 and 71% for DSC MRI (133). Specifically, they noted that the most accurate method for detection of recurrence was a combination of ^99m^Tc-MDM-SPECT/CT and DSC MR; however, the target to non-target ratio of MDM outperformed normalized CBV in distinguishing between patients with stable vs. progressive disease (133). Nevertheless, the need to perform delayed imaging of 2–4 h after radiotracer injection may be a practical limiting factor for the clinical use of ^99m^Tc-MDM SPECT/CT (133).

In clinical practice, PET is preferred over SPECT due to its superior spatial resolution which results in increased sensitivity (134). However, SPECT scanners may be more widely available in certain countries and practice settings where access to PET and PET tracers is limited (131).

### FDG PET

**Figure 5:**
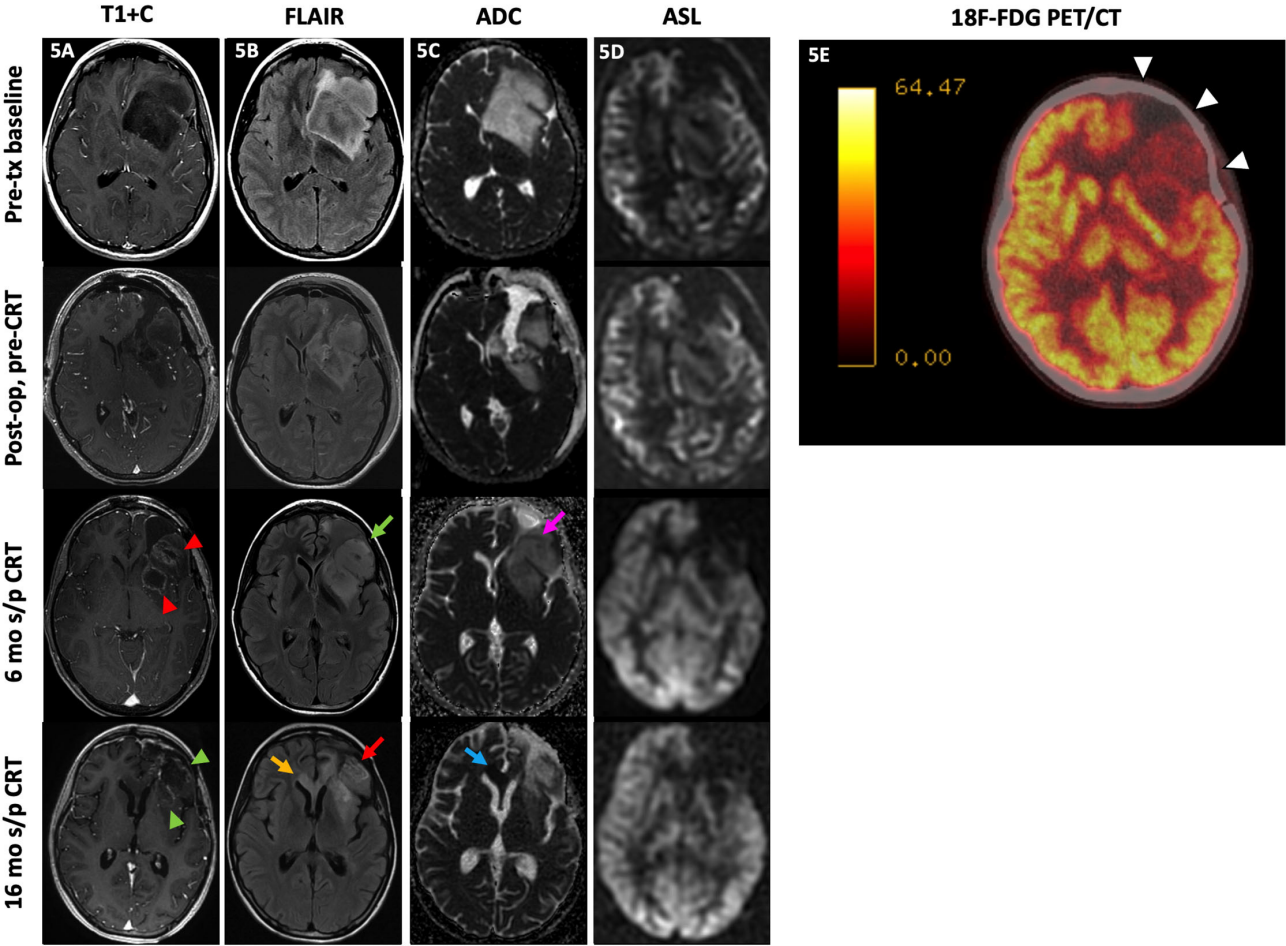
Treatment effect. A 17-year-old female with anaplastic astrocytoma (IDH wt, MGMT+) initially presented with a predominantly non-enhancing left frontal lobe mass [**(A)**, top row] with extensive locoregional mass effect, mild perilesional FLAIR hyperintensity [**(B)**, top row], and no substantial diffusion restriction [**(C)**, top row] or ASL hyperperfusion [**(D)**, top row]. After subtotal resection, there was no substantial enhancement [**(A)**, 2nd row] with mild residual FLAIR hyperintensity [**(B)**, 3rd row] without diffusion restriction [**(C)**, 3rd row] or ASL hyperperfusion [**(D)**, 4th row]. At 6 mo s/p CRT, new nodular enhancement was seen at the resection bed margins [**(A)**, 3rd row, red arrowheads] with increased expansile FLAIR hyperintensity [**(B)**, 3rd row, green arrow], mild diffusion restriction at the anterolateral aspect of the resection margin [**(C)**, 3rd row, pink arrow], and persistent lack of hyperperfusion on ASL images [**(D)**, 3rd row]. Subsequently, the patient underwent a ^18^F-FDG PET/CT a month later which showed hypometabolism throughout the left frontal resection cavity favored to represent treatment effect [**(E)**, white arrowheads]. Much later at 16 mo s/p CRT, the left frontal resection cavity showed decreased enhancement [**(A)**, 4th row, green arrowheads] with decreased FLAIR signal [**(B)**, 4th row, red arrow] at the areas of prior signal abnormality; however, there is a new focal area of FLAIR hyperintensity at the corpus callosum crossing midline [**(B)**, 4th row, yellow arrow] with diffusion restriction [**(C)**, 4th row, blue arrow] and no ASL hyperperfusion [**(D)**, 4th row]. These findings were consistent with delayed Psp/treatment effect at the left frontal resection cavity, which was supported by the PET/CT result, with a new area of probable recurrence at the corpus callosum. The patient did not undergo biopsy and passed away before additional follow-up imaging was obtained. ADC, apparent diffusion coefficient; ASL, arterial spin labeling; CRT, chemoradiotherapy; FDG, fluorodeoxyglucose; FLAIR, fluid-attenuated inversion recovery; mo, months; op, operative; PET/CT, positron emission tomography/computed tomography; s/p, status post;T1+C, post-contrast T1-weighted images; tx, treatment.

The use of ^18^F-FDG PET to distinguish residual or recurrent tumor from radiation necrosis relies on the concept that malignant cells and treated necrotic tissue have different rates of glucose metabolism. FDG, glucose analog in glycolysis, is trapped in the cell after phosphorylation by hexokinase; thus, FDG uptake is indicative of glucose metabolism (135). Malignant cells generally have high rates of aerobic metabolism while treatment-induced necrosis generally has decreased glucose metabolism (12, 135). Figure 5 demonstrates how FDG PET/CT may be used as a problem-solving tool. A 17-year-old patient with anaplastic astrocytoma post-resection and CRT developed new enhancement, expansile FLAIR hyperintensity, and diffusion restriction in the resection bed with subsequent PET/CT demonstrating hypometabolism, favoring treatment effect rather than recurrence. These suspicious MR findings in the resection bed eventually resolved and were fully appreciated 10 months later.

However, the FDG PET evaluation of brain tumors is limited by high physiologic uptake in normal gray matter. Several studies have reported a lower specificity for FDG PET than other functional and advanced imaging modalities with one study reporting a sensitivity of 81–86% and specificity of 40–94% (135–137). Additional pitfalls of FDG PET imaging are high rates of false positives from inflammatory processes and subclinical seizure activity (129). Furthermore, HGG may not demonstrate high FDG uptake if the degree of necrosis is severe; LGG may be obscured by the high background FDG uptake levels (136).

More recently, the use of PET/MRI combines the excellent soft tissue contrast of MR with the benefit of functional imaging provided by PET tracers (12, 54, 138). Simultaneous acquisition of MR or CT images with PET can also be performed in a reasonably timely manner in the clinical setting. Jena et al. performed a retrospective multiparametric study including the rCBV, mean ADC, Cho/Cr, and the maximum and mean target-to-background FDG uptake ratios and found that the AUC increased from 0.91 to 0.94 when the FDG PET parameter was combined with the mean ADC and Cho/Cr parameters for correctly classifying lesions as recurrence vs. treatment-induced necrosis (54).

### Amino Acid PET Radiotracers

Unlike FDG, amino acid (AA) PET is dependent upon the relative discrepancy in intracellular active uptake of amino acid radiopharmaceuticals *via* the large amino acid transporter system (LAT) between different cell types (139, 140). AA transport is upregulated in tumor cells compared to the normal brain parenchyma with AA transport occurring across intact BBB but low or absent in tissue with treatment-induced injury (54). In contrast to ceMR, BBB disruption is not a prerequisite for intratumoral localization of radiotracer (140, 141). Compared to FDG, AA radiotracers exhibit lower background physiologic uptake in the cerebral cortex and are expected to better differentiate between recurrence and treatment effect (142). Evidence in the literature suggests that the most commonly used AA PET tracers including, ^11^C-methionine (MET), O-(2-^18^F-fluoroethyl)-L-tyrosine (FET), and ^18^F-DOPA all demonstrate similar diagnostic performance (143).

^11^C-MET uptake is associated with increased cellularity as well as microvessel density and is increased in recurrence (113, 139). Deuschl et al. performed ^11^C-MET PET/MR and conventional MR on a cohort of 50 glioma patients and reported the sensitivities and specificities as 86% and 71% for conventional MRI, 97 and 74% for ^11^C-MET PET, and 97 and 93% for hybrid ^11^C-MET PET/MR (144). In a multicenter prospective trial comparing the diagnostic performance of ^11^C-MET with ^18^F-FDG in identifying recurrence, Yamaguchi et al. found that the diagnostic accuracy of ^11^C-MET-PET at 88% was superior to that of ^18^F-FDG-PET at 70% and reported the sensitivities of ^11^C-MET-PET and ^18^F-FDG-PET as 97 and 48%, respectively (142). Deng et al. conducted a meta-analysis of 17 articles and found that ^11^C-MET PET and DSC-MRI had comparable sensitivities of 87 and 88% as well as specificities of 81 and 85%, respectively (145).

^18^F-FET and ^18^F-FDOPA, developed subsequently to ^11^C-MET, have longer half-lives than ^11^C-MET, meaning they do not require the presence of an on-site cyclotron, which may not be available at all PET imaging centers (139). Bashir et al., among other studies, found a higher ^18^F-FET uptake in recurrent lesions and showed that ^18^F-FET uptake accurately differentiates treatment effect from recurrence with a sensitivity of 99% and specificity of 94% (146). Similarly, Galldiks et al. found that ^18^F-FET uptake is significantly lower in PsP than true progression (147). Puranik et al. found a sensitivity of 80% and specificity of 87.5% for a tumor-to-white matter cutoff value of 2.65 and suggested the use of ^18^F-FET PET as a problem-solving tool in the post-treatment setting. Kebir et al. noted that the mean and maximum tumor-to-brain (TBR) ^18^F-FET uptake ratios were increased in patients with enhancing lesions which presented 3 months after completion of CRT, suggesting its value in the evaluation of PsP (148). In a recent study by Werner et al., the mean TBR calculated from ^18^F-FET-PET showed the highest accuracy among static and dynamic ^18^F-FET uptake parameters (149). In the post-BVZ setting, George et al. showed that ^18^F-FET uptake decreases after BVZ therapy and only moderately correlates with contrast-enhanced T1 signal intensity; they also found that a high ratio of the ^18^F-FET uptake before and after treatment as well as an increased correlation between ^18^F-FET uptake and contrast-enhancing signal were associated with poor PFS and OS (150). The advantages of ^18^F-FET-PET include the lack of physiologic uptake in the striatum and relative ease and lower cost of synthesis (140, 151), whereas its limitations include non-specific tracer uptake secondary to slow renal elimination, lack of protocol standardization, and the potential need for delayed imaging (140).

Like ^18^F-FET, ^18^F-FDOPA is also transported across intact BBB and detects the extent of tumor beyond contrast enhancement in conventional MR (54). ^18^F-FDOPA is known to have a higher sensitivity for evaluation of LGG (54). Fraioli et al. demonstrated that the volume of residual tumor as characterized by ^18^F-FDOPA-PET is larger for HGG than for LGG compared with the tumor extent as depicted by conventional MR; ^18^F-FDOPA-PET was also able to detect recurrent disease in non-enhancing regions on ceMR (141). Herrmann et al. demonstrated that both visual assessment and semiquantitative assessment of ^18^F-FDOPA uptake with the mean and maximum standardized uptake values (SUV_mean_ and SUV_max_) values were highly accurate (82% for visual and 77–82% for semiquantitative assessment) and were significant predictors of PFS (152). Karunanithi et al. showed that in comparison to ^18^F-FDG, ^18^F-FDOPA performed higher in sensitivity and accuracy but not specificity (136). They reported the sensitivity, specificity, and accuracy for detecting tumor recurrence as 48, 100, and 61% for ^18^F-FDG-PET/CT and 100, 86, and 96% for ^18^F-FDOPA-PET/CT, respectively (136). Jena et al. found that when advanced MR parameters from ceMR, DSC-MRI, DWI, and MRS were interpreted in conjunction with ^18^F-FDOPA-PET, the diagnostic performance of the visual qualitative assessment of recurrence achieved an accuracy, sensitivity, and specificity of 95%, 96%, and nearly 100% (54). Youland et al. also confirmed that when combined with ceMR, ^18^F-FDOPA-PET improved the sensitivity and specificity to 94 and 75% (137). A particular advantage of ^18^F-FDOPA-PET is the rapid scan time with uptake levels peaking between 10 and 30 min after injection while ^18^F-FET-PET may require delayed phase imaging, which may be challenging for patients who have difficulty tolerating the exam (141). However, ^18^F-FDOPA-PET demonstrates physiologic uptake in the corpus striatum which could potentially obscure lesions close to the basal ganglia (153). Furthermore, the radiotracer is difficult to synthesize and may be limited in its availability (143). In a systematic review of the overall utility of AA PET in the post-BVZ assessment of treatment response, Hughes et al. found that AA PET is particularly useful in monitoring treatment response to BVZ in HGG patients (139). Additionally, ^18^F-FET-PET was able to diagnose BVZ treatment failure earlier than conventional MR (139).

## Treatment-Response-Assessment Maps (TRAMs)

Delayed-contrast MRI is a methodology that involves subtracting conventional T1W imaging from delayed T1W imaging to create treatment-response-assessment maps (TRAMS) (154). TRAMS has been shown to produce high resolution maps which show clear differentiation between tumoral and non-tumoral tissues in brain tumor patients. In this technique, ceT1W imaging is acquired at 3–5 min and at >1 h (60–105 min) after conventional injection of a contrast agent (154, 155). Regions in the TRAMS which show efficient clearance of contrast from the tissue were found to correlate with morphologically active tumor while regions of contrast accumulation were noted to consist of non-tumor tissues; regions which cleared the contrast on delayed phase images may consist of intact vessels while areas which retained contrast may consist of damaged vessel lumens in stages of necrosis (154, 156). Zach et al. found 100% sensitivity and 92% PPV in distinguishing areas of active tumor from non-tumoral abnormal tissues (154). Daniels et al. applied this technique to evaluate the response to BVZ in patients with recurrent HGG and found that the TRAMs technique achieved a 100% sensitivity, 87.5% specificity, 77.8% PPV, and 100% NPV, which outperformed both conventional T1W imaging and DSC-MR (157). However, an inevitable disadvantage of this technique is the requirement of scanning the patient again more than 1 h after contrast injection, which may limit its feasibility in clinical practice (154).

## Experimental and Emerging Imaging Techniques

Several experimental imaging methodologies have shown promise in supplementing the array of advanced and functional imaging techniques currently available for distinguishing recurrence and treatment effect. Specifically, these include amide proton transfer (APT) and sodium MRI (Na-MRI). APT, an emerging technique that relies on the chemical exchange saturation transfer (CEST) that provides signal intensity through the exchange of amide protons and bulk water proton, indirectly measures the mobile peptides and proteins within tumor cells as compared to normal tissue (158, 159). Several recent studies confirm that APT signal intensity is consistently higher in patients with disease recurrence and that APT can differentiate recurrence from treatment effect, further improving the diagnostic accuracy of other advanced MR imaging techniques such as PWI and PET (158–161). In a multiparametric evaluation of 30 glioma patients using ADC, rCBF, MRS ratios, and APT-weighted (APTw) effect, Liu et al. found that recurrent tumors demonstrated a substantially higher APTw as well as rCBF and that a combined use of APTw and rCBF achieved a higher diagnostic accuracy than either alone (AUC of 0.93 for the combined technique vs. 0.87 and 0.9 for APTw and rCBF alone, respectively) (52). Na-MRI takes advantage of the disruption in the sodium-potassium pump and sodium channels in tumor cells to generate different MR signals which arise from intra- and extracellular Na ions and have shown promise in evaluating tumor response (162–164). Several studies have shown increased Na concentrations in areas of tumor and necrosis when compared to the contralateral normal-appearing brain parenchyma although further investigations are need to determine if Na concentrations are reliably different between recurrent tumor and treatment-related necrosis (163, 164).

These types of multiparametric investigations have led to the advent of radiomics, the use of advanced computational methods to quantitatively identify and evaluate clinically relevant characteristics in treated gliomas that are too complex for the human eye to appreciate (165–167). For instance, Cai et al. were able to create a stratification model which integrated a set of radiomic features extracted from the pretreatment MRI of each patient and relevant clinical factors to predict which patients would benefit from BVZ therapy, with the model achieving AUCs of 0.91 and 0.83 in the validation data set (166). Revisiting their earlier work on distinguishing between vasogenic edema and peritumoral infiltrative signal with PWI and MRS, Artzi et al. employed a radiomics patch-based analysis and were able to classify non-enhancing perilesional signal into tumor and non-tumor areas in 102 patients with HGG (167). As an example of the use of deep learning in the detection of recurrent disease, Bacchi et al. used convolutional neural networks to construct models based on DWI, ADC, FLAIR, and post-contrast T1 sequences and found that the model based on DWI achieved an accuracy of 73% while the model based on both DWI and FLAIR achieved an accuracy of 82% (168).

## Use of Advanced Imaging in Specific Treatment Scenarios

Specific treatment scenarios also influence the utility of advanced imaging in post-treatment glioma patients. In the post-BVZ setting, Petrova et al. created a model with machine learning using DSC and ADC to identify patients who were highly likely to progress vs. those who were not likely to progress within 6 months after BVZ therapy (169). Stadlbauer et al. showed that rCBV itself was not able to distinguish PsR from true disease response post-BVZ therapy as brain perfusion decreased in both abnormal and contralateral normal appearing brain parenchyma (170).

Immunotherapy strategies against gliomas include specific peptide vaccines, immunotoxin therapy, immune checkpoint inhibitors, dendritic cell (DC) therapy, and chimeric angigen receptor T-cell (CAR-T) immunotherapy (8, 171). Immunotherapies may produce an inflammatory response that leads to increased contrast enhancement and vasogenic edema, mimicking progression (8, 171). Vrabec et al. noted significantly higher maximum rCBV ratios in patients with progression than treatment effect in the post-DC immunotherapy setting; the minimum ADC values in the contrast-enhancing regions were the lowest in the group of examinations obtained before definite evidence of progression in patients who eventually progressed (76). Qin et al. evaluated a small group of GBM patients receiving immune checkpoint blockade therapy and noted that the stabilization and improvement in the voxels with low ADC values in areas of FLAIR signal abnormality were predictive of therapeutic benefit (42). In a group of 22 GBM patients receiving DC immunotherapy, Cuccarini et al. found that the relative ADC value was predictive of response to immunotherapy as well as survival (171). In a group of children with diffuse intrinsic pontine gliomas, Ceschin et al. noted that serial parametric response mapping of ADC values following peptide-based vaccination may help distinguish progression from PsP (172). Efforts are ongoing to incorporate immunotherapy-related considerations into the RANO criteria (iRANO) to improve response assessment and clarify clinical guidelines for glioma patients in immunotherapy trials (173).

## Discussion

Since recognition of the limitations of conventional ceMR, substantial developments in advanced MR and functional imaging techniques have dramatically improved the overall diagnostic performance of neuroimaging in discriminating recurrence from treatment-related injury. These advanced MR and functional imaging techniques provide different but complementary information to conventional MR. Despite decades of reliance primarily on conventional MR in the post-treatment assessment of gliomas, ceMR only detects BBB disruption, which is a common pathophysiologic endpoint in recurrent glioma as well as cytotoxic injury after CRT. Increased signal on DWI and low ADC values reflect increased lesional cellularity while all three types of PWI discussed in this review are indicators of neovascularization and angiogenesis associated with glioma proliferation. Functional imaging with SPECT and PET tracers detects the increased metabolic requirements of tumor cells, such as glucose and amino acids, in contrast to decreased cellular metabolism in the setting of treatment-related necrosis.

While there is no consensus in the literature on a single or even a specific permutation of imaging modalities to best distinguish residual and recurrent disease from treatment effect, evidence in the literature overwhelmingly supports using advanced MR imaging and functional imaging in conjunction with conventional MR, with numerous multiparametric studies which used hybrid techniques achieving higher sensitivities, specificities, and accuracies than single modality methodology. Pellerin et al. and Beppu et al. investigated the combination of ASL imaging with PET. Pellerin et al. noted that while ^18^F-FDOPA-PET was highly sensitive (94%) for detecting recurrence, ASL was highly specific (up to 100%), with the two imaging modalities complementing each other (119). Beppu et al. found that while ASL and ^11^C-MET-PET uptake correlated at all time points status post-BVZ therapy, ^11^C-MET-PET uptake provided superior accuracy for the prediction of patients with long PFS. Hojjati et al. investigated the combination of ^18^F-FDG-PET/MRI, ^18^F-FDG-PET/CT, and DSC MRI, and concluded that although accuracy improved using PET/MRI compared to PET/CT, the combination of PET/MRI and PWI resulted in the best diagnostic performance overall. In a multiparametric study of MRS, DWI, and PWI parameters at 3T, Di Costanzo et al. found that the discrimination accuracy of MRS at 79% increased to 86% when ADC values in addition to MRS metabolite ratios were considered; a further increase in accuracy to 97% was achieved with the addition of rCBV (87). Cha et al. employed a multiparametric histogram analysis technique with rCBV and ADC values of indeterminate enhancing lesions over time, concluding that subtraction histograms with a multiparametric approach was more accurate than diagnoses based on the uniparametric approach, with an AUC of 0.88 for the multiparametric approach vs. 0.80 for the uniparametric approach (70). Anwar et al. performed a multiparametric voxel analysis with the goal of identifying voxel characteristics that may predict subclinical disease recurrence, noting that voxels with disease progression are significantly different in ADC, FA, and Cho/NAA values than voxels that were stable (174). Recently, Kim et al. created multiparametric spatiotemporal habitats by dividing enhancing lesions into three spatial habitats using clustering of voxel-wise ADC and rCBV values and assessed temporal changes in these habitats over two consecutive studies (175). They found that the strongest predictor of recurrence was an increase in the hypervascular cellular habitat which is characterized by low ADC and high rCBV (175).

Despite the lack of consensus on the most useful modality for imaging treated gliomas, a few imaging modalities have shown significantly lower diagnostic performance in comparison to other advanced MR and functional imaging techniques. In particular, multiple studies have reported the sensitivity and specificity of conventional MR alone around 60–70% (26, 137). ^18^F-FDG-PET alone has a reported sensitivity of ~40–50% (135, 136). Additional imaging techniques, such as ^201^Tl-SPECT, have fallen out of favor in current clinical practice due to the more appealing characteristics of ^99^Tc SPECT tracers which result in a lower radiation dose and newer PET tracers which have higher spatial resolution than SPECT tracers (40, 128).

Familiarity with the common challenges associated with studying the imaging of treated gliomas is crucial to inform future investigations. Histopathologic correlation is often considered the gold standard against which imaging findings are correlated; however, because of the inherent tumor heterogeneity of treated gliomas, histopathologic correlation can be complicated by inhomogeneous tissue sampling, sampling error, or inadequate volume of tissue samples to make a diagnosis (176). Furthermore, the histopathologic diagnosis of a treated lesion may not be straightforward and neuropathologists may not be able to reach a clear consensus (97). The RANO criteria addresses PsP as occurring primarily within the 3-month time period after CRT completion; however, delayed PsP may occur far later and may be more easily overlooked due to the time frame for PsP provided by the RANO criteria (14, 15). Additionally, variation in results in the literature are related to non-standardization in image acquisition, differences in thresholds used between institutions, small sample sizes, and variable post-processing methods, all of which limit the reproducibility of study results (2). In addition to multiparametric approaches, many investigations in recent years have trended toward more voxel-based, semiquantitative, and quantitative techniques as well as the use of machine learning models to improve reproducibility and increase standardization.

## Conclusion

This review offers a comprehensive overview of the current spectrum of conventional and advanced MR imaging with a brief exploration of promising experimental imaging techniques in the differentiation between glioma recurrence and treatment effect. While there is not a single modality or even a specific combination of modalities that is considered most useful overall, a helpful approach may be to combine techniques with high sensitivity with other modalities that have high specificity. Additionally, if the specific clinical indication is to screen for recurrence on surveillance evaluations, perhaps a modality or combination of modalities with higher sensitivity would be preferred to avoid a missed detection of recurrent disease. The multiparametric approach to imaging has the potential to triage patients with indeterminant enhancing lesions after CRT and accurately determine which patients may not require an invasive biopsy or repeat resection as well as guide clinical decision-making in a more timely manner than *via* serial follow-up imaging. The integration of multiparametric imaging parameters with independent clinical indicators through radiomics has great potential for the development of more personalized treatment protocols and improved prognostication that is especially pertinent given the limited survival of glioma patients.

## Author Contributions

AL: manuscript writing, image acquisition, editing, and reviewing. MI: idea generation, manuscript writing, image acquisition, editing, and reviewing. Both authors contributed to the article and approved the submitted version.

## Conflict of Interest

MI: consulting fees and stock options from Octave Bioscience, Inc., and consulting fees from Hanalytics Pte Ltd. However, these entities are not relevant to the submitted work. The remaining author declares that the research was conducted in the absence of any commercial or financial relationships that could be construed as a potential conflict of interest.

## Publisher's Note

All claims expressed in this article are solely those of the authors and do not necessarily represent those of their affiliated organizations, or those of the publisher, the editors and the reviewers. Any product that may be evaluated in this article, or claim that may be made by its manufacturer, is not guaranteed or endorsed by the publisher.
